# 

**Published:** 1905-06

**Authors:** 


					﻿Torpedo fop the Octopus.—'[Editorial.],
■■	: r t •	7	• , t r
; JUNE, 1905.
No. 12f
TEXAS
Medical Journal.
Subscription, $i.oo a Year, in Advance.
Single Copies, io Cents.
PUBLISHED AT AUSTIN, TEXAS, BY
JR F. E. DANIEL, M. D.,
Proprietor.
PRINTED BY THE VON BOECKMANN-JONE8 CO.
Entered at the Poetoffice at Austin, Texas, as Hecond-olass Mall Matter.
				

